# Characterization of *ETFDH* and *PHGDH* Mutations in a Patient with Mild Glutaric Aciduria Type II and Serine Deficiency

**DOI:** 10.3390/genes12050703

**Published:** 2021-05-08

**Authors:** Amanat Ali, Nahid Al Dhahouri, Fatmah Saeed Ali Almesmari, Waseem Mahmoud Fathalla, Fatma Al Jasmi

**Affiliations:** 1 Department of Genetics and Genomics, United Arab Emirates University, Abu Dhabi P.O. Box 15551, United Arab Emirates; amanat.a@uaeu.ac.ae (A.A.); 202090177@uaeu.ac.ae (N.A.D.); 201505025@uaeu.ac.ae (F.S.A.A.); 2 Department of Neurology, Sheikh Shakhbout Medical City, Abu Dhabi P.O. Box 11001, United Arab Emirates; wfathalla@seha.ae; 3 Department of Pediatrics, Tawam Hospital, Al Ain P.O. Box 15551, United Arab Emirates

**Keywords:** electron transfer flavoprotein dehydrogenase, GA-II, 3-phosphoglycerate dehydrogenase, serine deficiency, whole exome sequencing, Syria

## Abstract

Glutaric aciduria type II (GA-II) is a rare autosomal recessive disease caused by defects in electron transfer flavoprotein (ETF), ultimately causing insufficiencies in multiple acyl-CoA dehydrogenase (MAD). 3-phosphoglycerate dehydrogenase (3-PHGDH) deficiency, is another rare autosomal disorder that appears due to a defect in the synthesis of L-serine amino acid. Several mutations of *ETFDH* and *PHGDH* genes have been associated with different forms of GA-II and serine deficiency, respectively. In this study, we report a unique case of GA-II with serine deficiency using biochemical, genetic, and in silico approaches. The proband of Syrian descent had positive newborn screening (NBS) for GA-II. At two years of age, the patient presented with developmental regression, ataxia, and intractable seizures. Results of amino acid profiling demonstrated extremely low levels of serine. Confirmatory tests for GA-II and whole exome sequencing (WES) were performed to determine the etiology of intractable seizure. Sequencing results indicated a previously reported homozygous missense mutation, c.679 C>A (p.Pro227Thr) in the *ETFDH* gene and a novel missense homozygous mutation c.1219 T>C (p.Ser407Pro) in the *PHGDH* gene. In silico tools predicted these mutations as deleterious. Here, the clinical and biochemical investigations indicate that *ETFDH*:p.Pro227Thr and *PHGDH*:p.Ser407Pro variants likely underlie the pathogenesis of GA-II and serine deficiency, respectively. This study indicates that two rare autosomal recessive disorders should be considered in consanguineous families, more specifically in those with atypical presentation.

## 1. Introduction

Inherited metabolic diseases (IMD), also known as inborn errors of metabolism (IEM), include a diverse class of genetic disorders influencing metabolism. Glutaric aciduria type II (GA-II), also defined as multiple acyl-CoA dehydrogenase deficiency (MADD), is a rare IEM (MIM #231680), clinically categorized into three distinctive forms: neonatal-onset forms with and without congenital anomalies, and a mild or late-onset form [1,2]. Neonatal onset form is more lethal and generally presents with hypoglycemia, hypotonia, metabolic acidosis, and death in most cases. Additionally, laboratory examinations may show elevation of ammonia, liver enzymes, and cardiomyopathy [3,4].

GA-II, an autosomal recessive disease, appears due to defects in electron transfer flavoprotein (ETF) or ETF dehydrogenase (ETFDH), ultimately causing insufficiencies in MAD [5]. Variants of *ETFA*, *ETFB*, or *ETFDH* are mostly associated with GA-II [6]. Studies have also reported the involvement of certain variants of FAD synthase gene (*FLAD1*) in GA-II [7]. However, the onset of the disease and phenotypic presentations could differ based on the position and nature of the variants. Null variants in the *ETFDH* generally produce a complete loss of protein function or expression. In contrast, missense variants produce only a partial loss of enzyme activity and often lead to a mild clinical phenotype [8,9]. More than 190 different mutations have been reported in the *ETFDH* gene so far and were mostly observed in late-onset GA-II patients [4]. The electron transfer flavoprotein-ubiquinone oxidoreductase (ETF-QO) protein, encoded by the *ETFDH* gene, is a 617 amino acid long protein present on the inner mitochondrial membrane. It is comprised of three discrete co-factor binding domains, which are involved in the binding of flavin adenine dinucleotide (FAD), ubiquinone (UQ), and a [4Fe4S] cluster [10].

Serine deficiency disorders are mainly associated to defects in the metabolism of L-serine. It is endogenously produced from 3-phosphoglycerate (3-PG), a glycolytic intermediate, due to the sequential involvement of three enzymes: 3-phosphoglycerate dehydrogenase (3-PGDH or PHGDH), 3-phosphohydroxypyruvate aminotransferase, and phosphoserine phosphatase [11]. PHGDH deficiency (MIM# 601815) is a rare, autosomal recessive disorder, characterized biochemically by low concentration of serine in the plasma and cerebrospinal fluid (CSF) and clinically by serious neurological problems [12,13,14]. The clinical phenotype varies from nonspecific neurodevelopmental delays to Neu–Laxova syndrome (NLS), an extremely lethal disease [15,16]. NLS has been identified in over 100 cases so far [16]. NLS is mostly presented with several congenital defects and ectodermal abnormalities. Nonlethal serine biosynthesis deficiency includes a wide range of disorders such as epilepsy, neurodevelopmental defects, and microcephaly [17,18]. Several pathogenic mutations in the *PHGDH* gene have been strongly associated with nonlethal forms of serine deficiency in different populations [19,20]. The PHGDH enzyme, encoded by the *PHGDH* gene, is a 533 amino acid long protein. It is comprised of four domains, the substrate-binding domain, the nucleotide-binding domain, the allosteric substrate-binding domain (ASB), and the regulatory binding domain (ACT) [21]. The ACT domain signifies the first letters in Aspartate kinase, Chorismate mutase, and TyrA.

In this study, we report the detailed phenotype, biochemical and clinical findings of a case of GA-II and serine deficiency in a consanguineous family with missense homozygous variants in *ETFDH* and *PHGDH* genes.

## 2. Materials and Methods

### 2.1. Ethical Consideration

This study was approved by the Abu Dhabi Health Research and Technology Committee, reference number DOH/CVDC/2020/1185 as per national regulations. The index patient was identified by the neurology, metabolic and genetic team at Sheikh Shakhbout Medical City and Tawam Hospital, Abu Dhabi, for clinical evaluation and follow-up. After obtaining informed consent, blood samples from the index patient, her parents, and her sibling were collected on CentoCard (Centogene AG, Germany).

### 2.2. Biochemical Analysis

Quantitative acylcarnitines were measured from the plasma using tandem mass spectroscopy [22]. Plasma and cerebrospinal fluid (CSF) amino acid profiles were measured using high-performance liquid chromatography (HPLC) [23]. Urine organic acids were analyzed from the urine sample by using a gas chromatography-mass spectrometry approach [24]. Skin biopsy was taken from the patient for fibroblast culture and was cryopreserved immediately. Fatty Acid Oxidation Probe assay was performed on skin fibroblast using tandem mass spectrometry.

### 2.3. DNA Extraction and Whole Exome Sequencing

DNA was extracted from CentoCard (Centogene AG, Germany) using QIAsymphony DNA Investigator Kit (Qiagen, CA, USA), according to the manufacturer’s instructions. The Quality and quantity of DNA samples were checked using NanoQuant plate (TECAN, Mannedorf, Switzerland). Whole-exome sequencing (WES) for the index patient and parents was performed as a service at Centogene AG, Germany. Briefly, the exome capture was carried out using the Twist Human Core Exome Plus kit (Twist Bioscience, CA, USA), and the obtained libraries were consolidated, indexed, and target enriched. The generated libraries were sequenced using the Illumina platform, Novaseq 6000 sequencer to obtain a minimum 20x coverage depth for >98% of the targeted bases. Initially, the sequencing reads were transformed into normal Fastq format and were processed through an in-house maintained pipeline for the examination of WES data. Borrows wheeler aligner (BWA) software with maximum exact match (MEM) algorithm was used to align the short reads to the GRCh37 (hg19) build of the human reference genome. These alignments were further transformed to binary BAM file format. Subsequently, variant calling was carried out on the secondary alignment files using GATK HaplotypeCaller, freebayes, and samtools. Annovar and in-house established bioinformatics tools were used for variants annotation. Alignments were performed and visually verified by using Integrative Genomics Viewer v.2.38 and Alamut v.2.4.5 (Interactive Biosoftware, Rouen, France). The variants were further analyzed to classify those pertinent to the patient phenotype based on the recommendations of the American College of Medical Genetics (ACMG).

### 2.4. In Silico Analysis

The disease-causing or deleterious effect of missense SNPs was evaluated using different independent in silico tools: SIFT (Sorting Intolerant from Tolerant) [25] and PolyPhen-2 (Polymorphism Phenotyping v2) [26]. Furthermore, predictSNP2 [27] was also performed to determine the functional impact of SNPs using four databases (DANN, FATHMM, Funseq2, and GWAVA). Multiple sequence alignment (MSA) was carried out to determine the conservation of amino acids at the specific studied site and to calculate Jensen-Shannon Divergence (JSD) scores [28]. Briefly, amino acid sequences of ETFDH and PHGDH proteins from *Homo sapiens* (human), *Pan troglodytes* (chimpanzee), *Mus musculus* (house mouse), *Rattus norvegicus* (Norway rat), *Canis lupus* familiaris (dog), *Equus caballus* (horse), *Bos taurus* (bovine), *Xenopus tropicalis* (frog), and *Gallus gallus* (chicken), were obtained from NCBI RefSeq and aligned using ClustalW [29]. Aligned sequences in FASTA format were used to calculate JSD scores. For homology modeling, the protein sequences of the human mitochondrial ETF-QO and PHGDH were obtained from UniProt (Accession number: Q16134, and O43175). Modeling was performed using the following structures from the protein data bank (PDB): ETF-QO–PDB ID: 2GMH [10]; PHGDH–PDB ID: 1YGY [21]. Homology models were produced using SWISS-MODEL [26] and confirmed by I-TASSER (Iterative Threading ASSEmbly Refinement) [30]. The generated models were evaluated and visualized in PyMOL [31].

## 3. Results

### 3.1. Case Presentation

**Figure 1:**
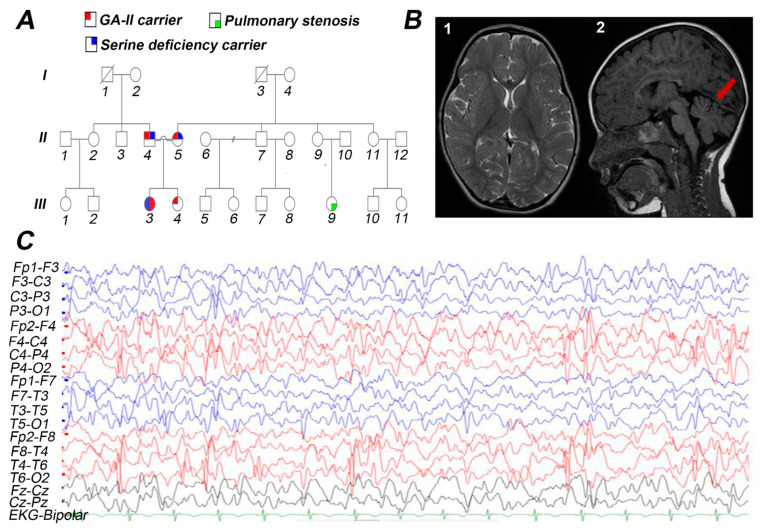
The detailed pedigree, EEG, and brain MRI of the index patient performed at 2.5 years of age. (**A**) Family pedigree of three generations affected with GA-II and serine deficiency. Male and female are represented by squares and circles, respectively. The proband is represented with a filled circle. (**B-1**) Axial T2 shows normal basal ganglia; (**B-2**) Sagittal FLAIR signifies mild cerebellar atrophy shown in red arrow. (**C**) EEG shows diffuse slow background with prominent right occipital spike and slow-wave discharges.

A female proband was born at full term to healthy consanguineous Syrian parents in the United Arab Emirates after a normal pregnancy. The baby was delivered via spontaneous vaginal delivery (SVD). The weight of an infant at the time of birth was 2700 g. The infant was discharged from the hospital on the postnatal second day since no anomalies were observed during the physical examination. Proband was referred to a metabolic physician at six days of age after the newborn screening (NBS) results reported to be positive for GA II. The first acylcarnitine test was reported non-conclusive (Table 1). However, a follow-up acylcarnitine report was negative. At 1 year and 7 months, a squint was observed, which was later improved by eyeglasses and eye patches. The proband developed her first seizure attack at 25 months of age. The semiology was brief up-rolling of the eyes with eyelid myoclonus, but no other convulsive activity was observed. She was started on levetiracetam and oxcarbazepine, but her seizures remained persistent. Her first electroencephalogram (EEG) report was normal, but her second EEG reported focal occipital spikes (Figure 1C). The parents sought a second opinion where her anti-seizure medications were changed to a combination of clonazepam, lamotrigine, and valproic acid, which resulted in a period of full control of her seizures. Brain magnetic resonance imaging (MRI) performed at 2.5 years of age showed cerebellar atrophy (Figure 1B). At 3 years of age, the proband presented with recurrent seizures and regression of speech, balance, and fine motor skills. Seizure episodes were brief 1–2 s of eye-blinking, and head or full body drop, suspicious for atypical absence or Myoclonic Astatic Epilepsy (MAE) of Doose. Her physical exam was remarkable for microcephaly, slurred speech, wide-based ataxic gait, and mild generalized hypotonia. The intractable seizure and neurodevelopmental regression, possibly aggravated by valproic acid, raised concern for a metabolic cause of her epilepsy, and thus comprehensive genetic epilepsy gene panel was ordered. Results identified a homozygous missense variant c.679C>A (p.Pro227Thr) in the *ETFDH* gene. This further strengthened the suspicion of valproic acid aggravating her neurodevelopmental regression. Therefore, valproic acid was withdrawn, after which her ataxia and unsteadiness improved significantly. She remained delayed in speech, fine motor skills, and her seizures remained intractable. At 4 years of age, she was referred to a metabolic center for confirmation of her GA-II, and WES was recommended as part of her workup. WES results identified homozygous variants *ETFDH*:c.679C>A (p.Pro227Thr) and *PHGDH*:c.1219T>C (p.Ser407Pro), related to GA-II and serine deficiency, respectively. The plasma and cerebrospinal fluid (CSF) amino acid profiling studies revealed a severe serine deficiency (Table 2). The proband was managed with L-serine 1500 mg per oral thrice a day (Table 2). As a result of initiating the amino acid therapy, a notable improvement in behavior and quality of life was observed, and seizures also disappeared. The plasma serine concentration increased from 42 µmol/L to 136 µmol/L during the treatment (Table 2). While the GA-II-related complications of this patient were managed with riboflavin 100 mg thrice a day, coenzyme Q10 (ubiquinone) 10 mg once daily, and levocarnitine 1000 mg twice a day. Dietary counseling was also provided to the patient specifically for a low-fat, and high carbohydrate diet.

### 3.2. Biochemical Studies

Biochemical results indicated normal levels of urine organic acids. Since the tests were not performed during the acute episodes of intercurrent illnesses, thus this could be the reason for the negative report of urine organic acids. Liver and kidney function tests were normal. Results of plasma acylcarnitines showed elevated levels of C3, C5-DC, C6, C8, C10, C12, C14, and C14:1 (Table 1). However, the results of the fatty acid oxidation probe assay of the proband did not demonstrate any abnormalities when compared to controls. This suggests that this could be a case of real riboflavin-responsive form, and the presence of riboflavin in the culture media of fibroblast could have masked the riboflavin-sensitive GA-II enzymes. Additionally, amino acid profiling of the patient exhibited abnormal results (Table 2). Importantly, significant deficiency in plasma level of serine (42 µmol/L) was observed along with a mildly reduced concentration of glycine (96 µmol/L). CSF sample exhibited extremely reduced levels of serine (6 µmol/L) with a normal concentration of glycine (8 µmol/L); serine CSF/plasma ratio:0.14 (Ref > 0.2), which is consistent with the diagnosis of serine deficiency.

### 3.3. Molecular Findings

WES was performed to determine the etiology of intractable seizure. Variants were filtered based on the clinical observations of the patient, family history, suspected disease pathways, and minor allele frequency (MAF ≤ 0.05). These variants were predicted as deleterious or benign by using bioinformatics tools. Based on the ACMG classification, two variants of uncertain significance (VUS) were detected (Table 3). Of the two, *ETFDH* c.679C>A (p.Pro227Thr), a missense variant, had already been reported in ClinVar. However, to the best of our knowledge, *PHGDH* c.1219T>C (p.Ser407Pro), another missense variant, has not been reported in any database. Both variants were detected in the proband in a homozygous state and in the parents in a heterozygous state. However, only the *ETFDH* c.679C>A variant was detected in her healthy sibling in a heterozygous state. *PHGDH*:p.Ser407Pro has contradictory predictions of pathogenicity. This is primarily due to the low scores of PROVEAN, PANTHER, and FATHMM (Table 3). However, here the clinical and biochemical information of the index patient is suggestive for pathogenicity of these variants.

### 3.4. Bioinformatics Analysis

**Figure 2:**
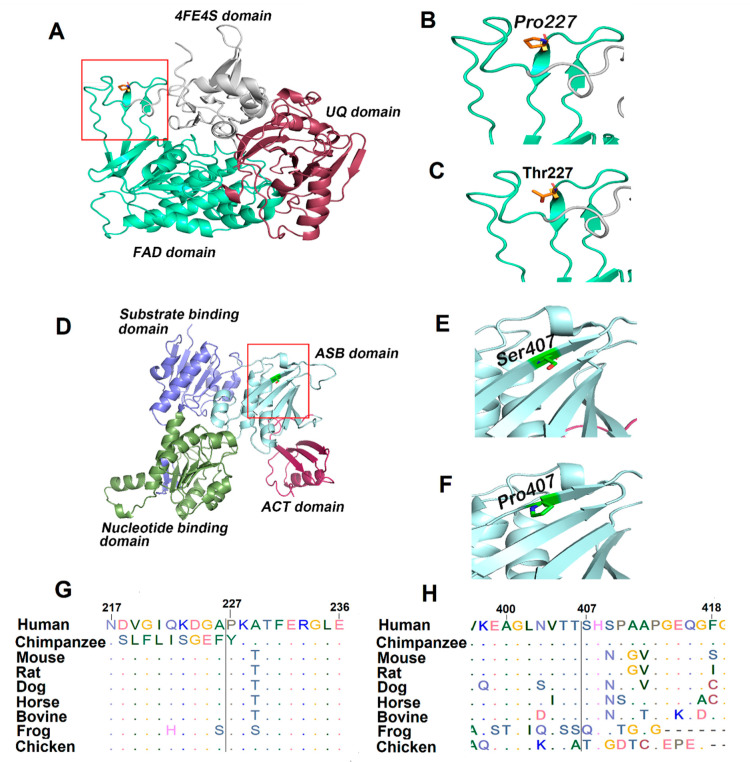
The generated homology models of ETFDH and PHGDH. The functional domains of ETFDH and PHGDH are demonstrated in cartoon representation, and the amino acid is represented with a stick representation. The boxed region shown red in A and D is enlarged in the successive images. (**A**) Modeled structure of ETFDH; (**B**) Wild type Pro227; (**C**) Mutant Thr227. (**D**) Modeled structure of PHGDH; (**E**) Wild type Ser407; (**F**) Mutant Pro407. Multiple sequence alignment of twenty amino acids centered on the missense mutation obtained from different mammals. (**G**) ETFDH, P227T (c. 679C>A); (**H**) PHGDH, S407P (c. 1219T>C).

Several bioinformatics tools were used to determine the pathogenic charge of detected missense variants. ETFDH and PHGDH protein sequences of different mammals, as shown in Figure 2G,H, were retrieved from NCBI and MSA was performed using ClustalW [23]. Pro227 and Ser407 residues of ETFDH and PHGDH, respectively, are located in conserved regions, as indicated by their respective JSD scores (Figure 2G,H, and Table 3). Thus, it is perceivable that their substitutions could likely affect the functions of ETFDH and PHGDH. The results of predictory algorithms also support this hypothesis (Table 3). Finally, to evaluate the effect of missense mutations on the protein functions, three-dimensional structures of wild type and the variant of human ETF:QO and PHGDH proteins were modeled (Figure 2A,D). The human ETF:QO protein sequence showed a sequence identity of 95% with porcine ETF:QO. Structural analysis indicated that the wild type Pro227 produces a perceptible kink in the structure. However, the introduction of polar residue Thr227 is likely to produce more flexibility (Figure 2B,C). The human PHGDH protein sequence exhibited 34.39% sequence identity with mycobacterium tuberculosis PHGDH. The wild type Ser407 is located on β-strand present in the ASB site of PHGDH. The substitution of serine to proline at the 407 position produced a perceptible kink in the β-strand (Figure 2E,F).

## 4. Discussion

This study reports a clinically diagnosed GA-II patient with serine deficiency in plasma and CSF and expands the clinical understanding of this disorder towards the onset of childhood intractable seizures and neurodevelopmental delays. Two missense variants *ETFDH*:p.Pro227Thr and *PHGDH*:p.Ser407Pro were identified in the proband. Biochemical and clinical findings indicate that these mutations likely underlie the pathogenesis of GA-II and serine deficiency.

Several variants of *ETFDH* have been associated with GA-II and are considered important indicators [8,32]. Sequencing results identified an already reported mutation (*ETFDH*:p.Pro227Thr) in the ClinVar as a VUS. Another variant of Pro227(p.Pro227Ser) has also been identified in two patients in a heterozygous state [33]. However, no functional studies have been performed to evaluate the clinical significance of these variants. Pro227, a conserved exposed residue, is present at the FAD-binding domain of ETF-QO. The co-factor FAD is essential for the appropriate protein folding, stability, and catalytic activity of the ETF-QO enzyme. Therefore, its substitution with Thr227 is likely to change the conformation adjacent to the FAD binding site and could disturb the binding stability of FAD, which is important for enzyme activation. Importantly, novel or infrequent variants may not be recognized by bioinformatics tools and can be considered as variants of unknown significance (VUS). Therefore, deep analysis and interpretation of genetic findings are crucial. Patients with a mild form of GA-II generally have normal acylcarnitine between the intercurrent illnesses, and for such cases, genetic testing is recommended as part of a positive newborn screening confirmatory workup. Several studies have shown that diagnosed patients of GA-II with missense mutations in the *ETFDH* gene often demonstrate improvement in their clinical symptoms upon receiving riboflavin therapy [7,34,35]. Such patients are described as riboflavin responsive MADD (RR-MADD). Riboflavin is a substrate that is eventually converted to FAD. This study also reports a riboflavin-responsive patient of GA-II with a missense mutation in the *ETFDH* gene.

This report highlights the importance of performing acylcarnitine and urine organic acid profiles during acute illness for the meaningful diagnosis of GA-II. Similarly, plasma and CSF amino acid profiling are equally important for the workup of patients with intractable seizures. Serine deficiency disorders are mainly neurological in nature and are caused by specific mutations in the *PHGDH* gene [36]. The essential role of *PHGDH* in L-serine synthesis has been reported, and results indicated that targeted disruption of the *PHGDH* gene in a mouse model produced severe neurodevelopmental defects [37]. Different onset forms (infantile, juvenile, and adult) of serine deficiency associated with specific mutations in the *PHGDH* gene have been reported [36]. Among these, the infantile form is more lethal and is characterized by congenital microcephaly, severe neurodevelopmental disorders, and intractable seizures. Interestingly, the patient described in this study had biochemical abnormalities similar to those observed in patients with severe phenotypes. The plasma and CSF serine levels of the proband were comparable to those found in severely affected infants (Table 2). Moreover, studies have indicated that phenotypic variability in serine deficiency disorders is mainly linked to the degree of PHGDH enzyme activity [16,38]. Here, we reported childhood phenotypes of PHGDH deficiency with microcephaly, intractable seizure, and developmental regression without hypomyelination. A homozygous missense mutation (p.Ser407Pro) of PHGDH was detected by WES. Ser407 is present at the allosteric substrate binding (ASB) site (Figure 2E,F). Different missense mutations in the ASB site of PHGDH have previously been associated with serine deficiency [11,38]. Therefore, it is conceivable that this conservative change where polar amino acid (Ser407) was substituted with hydrophobic amino acid (Pro407) at the carboxyl-terminal of the PHGDH could hinder enzyme activity and likely be the reason for low serine levels. It is worth mentioning that the *PHGDH* gene was not included in the previously ordered epilepsy gene panel. Thus, this gene should be prioritized for intractable seizure and included in a comprehensive epilepsy gene panel. Moreover, enzymatic activities of such rare diseases are not available on a clinical basis, and this creates an additional challenge in the diagnosis of patients with a rare disease.

Overall, we report a unique case of GA-II with significantly low levels of serine as well as a novel mutation (p.Ser407Pro) in the *PHGDH* gene. This study also describes that both variants of *ETFDH* and *PHGDH* segregate in an autosomal recessive mode of inheritance. It is important to consider genetic studies for GA-II patients with atypical presentation. Additionally, classifying pathogenic variants, such as those demonstrated in this study, will help in effective family counseling intended for genetic prevention.

## Figures and Tables

**Table 1:** Quantitative acylcarnitine profile of the patient.

Analyte	Result	Interpretation	Reference Range	Unit
Acetylcarnitine, C2	7.32	N	2.14–15.89	nmol/mL
Propionylcarnitine, C3	1.16	**H**	<0.55	nmol/mL
Iso-/Butyrylcarnitine, C4	0.38	N	<0.46	nmol/mL
Isovaleyrl-/2-Methylbutyrylcarnitine, C5	0.28	N	<0.38	nmol/mL
Glutarylcarnitine, C5:DC	0.07	**H**	<0.06	nmol/mL
Hexanoylcarnitine, C6	0.19	**H**	<0.14	nmol/mL
3-OH-hexanoylcarnitine, C6-OH	0.01	N	<0.08	nmol/mL
Octanoylcarnitine, C8	0.47	**H**	<0.19	nmol/mL
Octenoylcarnitine, C8:1	0.20	N	<0.48	nmol/mL
Decanoylcarnitine, C10	0.77	**H**	<0.27	nmol/mL
Decenoylcarnitine, C10:1	0.20	N	<0.25	nmol/mL
Dodecanoylcarnitine, C12	0.28	**H**	<0.18	nmol/mL
3-OH-dodecanoylcarnitine, C12-OH	0.03	N	<0.06	nmol/mL
Tetradecanoylcarnitine, C14	0.12	**H**	<0.11	nmol/mL
Tetradecenoylcarnitine, C14:1	0.20	**H**	<0.16	nmol/mL
3-OH-tetradecanoylcarnitine, C14-OH	0.01	N	<0.04	nmol/mL
Hexadecanoylcarnitine, C16	0.13	N	<0.36	nmol/mL
Hexadecenoylcarnitine, C16:1	0.04	N	<0.15	nmol/mL
3-OH-hexadecanoylcarnitine, C16-OH	0.01	N	<0.78	nmol/mL
Stearoylcarnitine, C18	0.05	N	<0.10	nmol/mL
Oleylcarnitine, C18:1	0.16	N	<0.25	nmol/mL

N: normal; H: High.

**Table 2:** Plasma and CSF levels of serine and glycine of the patient before and after the treatment with L-serine.

Amino Acid	Before Treatment	After Treatment	Reference Range (1–5 Years)	Unit
Plasma serine	42	184	115–169	µmol/L
Plasma Glycine	96	145	175–283	µmol/L
CSF serine	6	NP	56–103	µmol/L
CSF glycine	8	NP	6.8–15	µmol/L

CSF: cerebrospinal fluid; NP: not performed.

**Table 3:** In silico prediction and analysis of *ETFDH* and *PHGDH* variants detected in the patient.

Gene	Missense Substitutions	Zygosity	Polyphen	SIFT	PROVEAN	PANTHER	DANN	GWAVA	Funseq2	FATHMM	Frequency	JSD
***ETFDH***	c. 679C>A; p. Pro227Thr	Homozygous	0.989 D	0.01 D	−7.52 D	0.95 D	0.997 D	0.45 Unknown	4 D	0.991 D	0.000025	0.782
***PHGDH***	c. 1219T>C; p. Ser407Pro	Homozygous	0.736 D	0.041 D	−1.79 N	0.27 B	0.994 D	0.28 D	4 D	0.620 N	ND	0.766

Polyphen-2 (higher scores indicate pathogenicity), SIFT (lower scores indicate pathogenicity), PROVEAN (protein variation effect analyzer, the cut-off score was −2.5, values equal to or above this threshold suggest the mutation is deleterious), PANTHER (Scores equal or above 0.5 indicate the mutation is deleterious or diseased), DANN (deleterious annotation of genetic variants using a neural network) is a method for scoring the deleteriousness of the SNVs (single nucleotide variants) using deep neural network classifier. GWAVA (genome-wide annotation of variants) is a method for scoring the deleteriousness of the SNVs based on random forest. Funseq2 is a method for scoring the deleteriousness of the SNVs based on a weighted scoring system that integrates genetic, epigenetic, and gene expression information. FATHMM (functional analysis through hidden Markov models) is a method for predicting pathogenicity based on multiple sequence alignment of protein sequences and/or structures. JSD score is used to determine the conservation of amino acids (higher scores demonstrate more conservation. Allele frequency was from the exome aggregation consortium (ExAc). ND: no data; D: deleterious; B: Benign; N: Neutral.
